# Supplementary material for: Training load, sports performance, physical and mental health during the COVID-19 pandemic: A prospective cohort of Swiss elite athletes
Source: PLoS One. 2022 Dec 1;17(12):e0278203. doi: 10.1371/journal.pone.0278203 (PMC9714901; doi:10.1371/journal.pone.0278203)
Supplement: Attachment — Submitted filename: Review 20220624.docx [file pone.0278203.s002.docx]

Dear Dr. Tauqeer Hussain Mallhi

Thank you for providing me an opportunity to read the review PONE-D-21-35814 which was submitted to the PLOS ONE editorial office. I carefully read the manuscript entitled " Sports performance, mental health, physical health, and training load during the progression of the COVID-19 pandemic: A cohort study of Swiss elite athletes". The authors evaluated the relationship between COVID-19 pandemic and mental/physical health among the elite athletes.

Although the theme of this study is interesting, there are some concerns.

The main problem is the definition about “elite athlete” did not clarify in this study. Therefore, although the authors revealed the differences of the prevalence between this study and other studies, I can’t evaluate about this.

Besides, there are some insufficient descriptions in the result section.

For the above reasons, I recommend that this paper be accepted after major revision.

I really appreciate if you would understand and accept my opinion.

With best regards

Kohei Ashikaga, MD, PhD

**Comments to Author**

**GENERAL COMMENTS**

In this manuscript, the authors evaluated the relationship between COVID-19 pandemic and mental/physical health among the elite athletes. It was interesting that the value changed depending on the timing and the factors. However, the authors should clarify the definition of elite athlete in this study. Are they national team member? Although the authors revealed the differences of the prevalence between this study and other studies, it is difficult to evaluate about this.

**Major**

・P11 Line184-185, Figure 2a: Description about this part is insufficient. In this figure, there does not seem to have significant difference compared to before the lockdown. The authors need to show statistical numbers more specifically. And the authors need to show the meaning of dotted lines in the Fig. 2.

・P13 Line 201: How do you think about there is a difference between winter and summer sports?

・P15 Line 246: Suddenly the description about COVID-19 has begun. Please describe how to diagnose or assess the COVID-19 in the method.

**Minor**

P5 Line 109: The authors need to explain the abbreviation for FUP here

P14 Line 217-245: The authors need to indicate where these descriptions are related to the figure.
